# The Slope Safety, Heavy Metal Leaching, and Pollutant Diffusion Prediction Properties under the Influence of Unclassified Cemented Paste Backfill in an Open Pit

**DOI:** 10.3390/ijerph191912772

**Published:** 2022-10-06

**Authors:** Ke Chen, Qinli Zhang, Yunbo Tao, Kai Luo, Qiusong Chen

**Affiliations:** School of Resources and Safety Engineering, Central South University, Changsha 410083, China

**Keywords:** open pit, unclassified cemented paste backfill, slope safety simulation, heavy metal leaching, groundwater monitoring

## Abstract

Open-pit unclassified cemented paste backfilling (OPUCPB) methods have not only addressed the disposal problems of tailings but also eliminated geological hazards of high and steep open pit slopes and created conditions for ecological restoration of the open pit in the future. In this paper, slope safety simulations, heavy metal leaching, groundwater monitoring, and pollutant diffusion predictions were examined to evaluate the slope safety pattern and environmental protection enabled by OPUCPB. The results showed that: (1) The safety factor of the open pit slope was proportional to the height of OPUCPB operation. Under the condition of seismic force and seepage field, the safety factor of slope B was increased from 1.188 to 1.574 by OPUCPB. (2) The toxic and harmful components in tailings were significantly stabilized by the OPUCPB. Under the conditions of acid leaching and water leaching, the quality of the leaching solution met the requirements of the class III limit of groundwater (GB/T14848-2017). (3) The monitoring results of groundwater quality around the open pit showed that the OPUCPB had no effect on groundwater, and the water quality met the requirements of the class III limit of groundwater (GB/T14848-2017). (4) Considering the diffusion prediction of pollutants and groundwater under extreme conditions, it was found that the pollution process is slow, and the shortest time required for pollutants to reach the standard limit is 232 d at a distance of 50 m from the leakage point. Therefore, the influence of OPUCPB can be controlled, and this method can achieve improved reclamation of open pits and safety treatment of tailings. It was worth popularizing and applying in mining enterprises.

## 1. Introduction

The rapid development of the mining industry can provide a lot of raw materials to other industries, which can support growth of the economy and innovative technology [1]. When the stripping ratio of open-pit mining reaches an economically reasonable value, the open-pit mining is converted to underground mining. Therefore, open pits are left on the surface. According to China’s current land reclamation regulations, mining companies need to implement the principle of “whoever destroys, whoever reclaims”. Ultimately, mining enterprises need to restore and manage open pits. If the open pit remains on the surface, it will cause the risk of slope collapse [2].

Subsequently, tailings are produced in high quantity since they are the by-product of mineral processing. According to the literature, more than 25 billion tons of tailings have been produced in China, with an increased rate of 600 million tons of tailings per year [3,4,5]. Notably, cemented paste backfill has been used in many mining industries to address the risks of tailings, including environmental pollution, a large amount of land occupation, and tailing ponds failure risks [6].

Due to the numerous problems and dangers encountered while working in mines, many scholars and project managers have found a comprehensive solution to the above problems. That is, using the unclassified tailings cemented backfilling system to backfill the remaining tailings from the underground backfill to the open pit, known as open-pit unclassified cemented paste backfilling (OPUCPB). OPUCPB can efficiently solve the problem of tailings discharge in the dressing plant and eliminate the hidden dangers of geological disasters associated with high and steep open-pit slopes. Moreover, creating conditions for the future ecological restoration of open pits is an environmental protection project that benefits the country and the people. For example, the Shirengou ore mine used cemented paste backfill to backfill the open-pit, and it greatly improved the safety of the open-pit slope and underground mining activities [3].

However, because OPUCPB was a new technology applied in response to the stricter regulation of tailings ponds, no unified technical standard or specification has been developed. Optimized standard practices are necessary to ensure the safety and environmental protection of OPUCPB and provide experience that can be used by mines under similar conditions. The key impacts of OPUCPB on the safety of slopes, heavy metal leaching concentration, and diffusion of heavy metals in groundwater have been investigated.

The safety of slopes is a major engineering challenge and safety concern. Thus, many scholars investigated the influence of slope parameters on safety, which often relied on simulation software. For example, Wenwu Sun et al. used the JanBu method to digitally analyze the slope safety of an iron mine [7]. Furthermore, Jinhai Wang et al. optimized the iterative formula of the traditional JanBu method [8]. Hua-Ming Tian et al. used BUS and subset simulation methods to investigate the safety of reinforced slopes [9]. Peidong Su et al. used a two-dimensional limit equilibrium method and three-dimensional numeric simulation to predict the stability of a high slope in an open pit [10]. Among these simulation methods, Slide software and JanBu method based on the optimization non-arc method are widely used in slope safety studies.

The safety of the environment is another major problem, where including the diffusion and heavy metal leaching [11,12]. In order to evaluate the degree of environmental pollution by waste solid, water leaching, and acid leaching were all used as the main methods in heavy metal leaching research [13,14]. These methods including toxicity characteristic leaching procedures (TCLP), extraction procedure (EP), solid wastes-roll over leaching procedure (GB5086.1-1997), acetic acid buffer solution method (HJ/T300-2007) and so on [15,16]. In addition, many scholars used these methods to investigate the toxicity leaching properties of solid waste, the toxicity fixation capacity of new materials and the feasibility of reusing waste. The above methods can only study the concentration of heavy metal leaching from instantaneous solid waste but cannot study the degree of pollution diffusion in groundwater over time. To study the diffusion rate and degree of heavy metals in groundwater under varying temporal and spatial conditions, several scholars have developed methods of monitoring and forecasting [17,18]. For example, Dragana Adamovic et al. monitored the groundwater quality around porphyry copper mining areas and found that calcium and sulfate were good indicators for monitoring early groundwater pollution caused by mining [19]. Furthermore, Ruitao Jia et al. developed an optimized layout of groundwater monitoring [20]. Sudhakar Singha et el. adopted an efficient machine learning technique to predict the groundwater quality, which obtained accurate and reliable prediction results [18,21]. Hamid Kardan Moghaddam et al. used the Bayesian approach to predict groundwater pollution [22]. Although the prediction results of the above methods are accurate, the processes are complex and require sophisticated equipment. To predict the diffusion of heavy metals in groundwater simply and conveniently, an economical and reliable prediction model and one-dimensional hydrodynamic model were used to study the diffusion concentration of heavy metals in groundwater over time [23].

In this paper, the process of backfilling an open pit with cemented tailings is discussed. This study used Slide simulation software for slope safety factor calculations, and water leaching, and acid leaching methods were used to study the metal leaching concentration of unclassified tailings and backfill cement bodies. Three monitoring points were set around the open pit to monitor the concentration of heavy metals in groundwater at each point, and a one-dimensional stable flow one-dimensional hydrodynamic dispersion model was used to predict the concentration and diffusion of heavy metals.

## 2. Materials and Methods

### 2.1. Study Site

**Figure 1:**
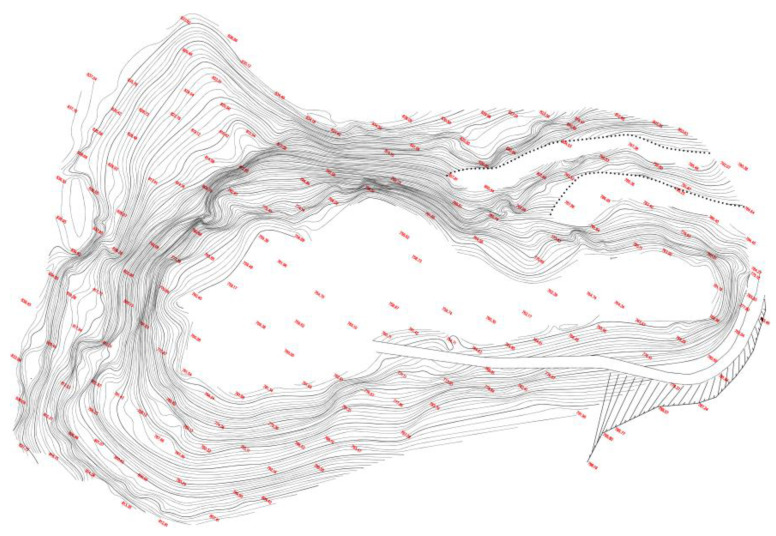
Original appearance of the open pit.

**Figure 2:**
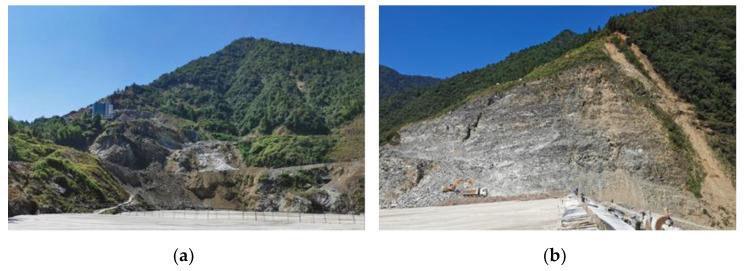
Engineering photos of the slopes: (**a**) A slope; (**b**) A slope.

The engineering case study of cemented paste backfill (CPB) was conducted in an open pit in China. In the early stage, the mining elevation was 780–890 m. When the open pit mining was finished, an abandoned open pit with a total area of 24,000 m^2^ was left. The upper side of the open pit was 307 × 179 m, and the bottom size was 149 × 94 m. The final slope angle of the open pit was large, about 63° in the south and 66° in the north, and the maximum slope height was more than 130 m, which can be categorized as a high and steep open slope. Moreover, cracks and rolling stones were observed on high and steep slopes on both sides. Figure 1 shows the original appearance of the open pit. In addition, the engineering photos are shown in Figure 2.

### 2.2. Backfilling Implementation

To eliminate geological hazards of high and steep open-pit slopes and solve the problem of tailings discharge, the unclassified tailings CPB was used in the open pit. The designed height of the CPB was 80 m (760–840 m). The height of backfill in the first stage was 24 m (760–786 m), and the cement/tailings ratio was 1:14. The second stage was 14 m (786,800 m), and the cement/tailings ratio was 1:16. The third stage was 8 m (800–808 m), and the cement/tailings ratio was 1:20. The fourth stage was 32 m (808–840 m), and the cement/tailings ratio was 1:20.

### 2.3. Experiments

#### 2.3.1. Physical Tests

(1) Physical properties of rock and CPB sample

**Figure 3:**
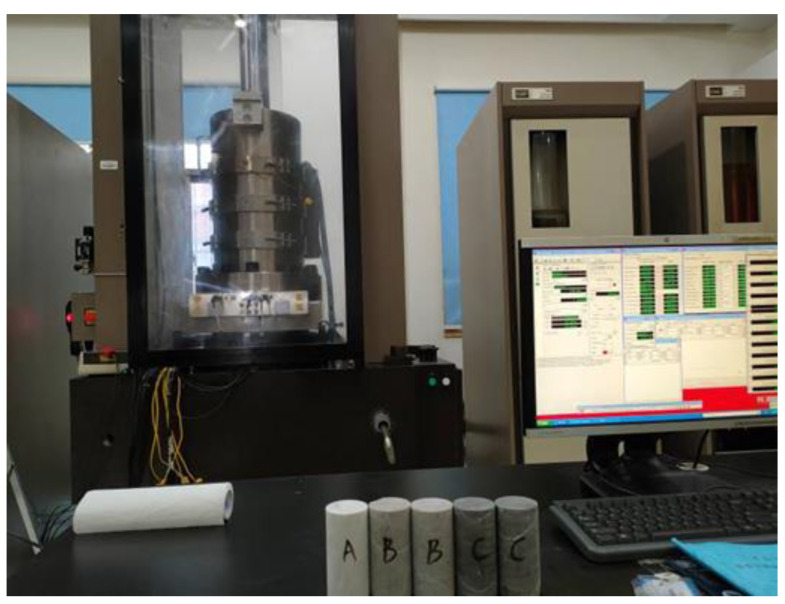
Rock triaxial test system (MTS 815).

A rock triaxial test system (MTS 815) was used to test the physical properties of the CPB sample and the rock slope, including the uniaxial compressive strength, elasticity modulus, Poisson ratio, osmotic coefficient, and tensile strength. Every property was tested three times, and the final result is reported as the average of the three test results. The test machine is shown in Figure 3, and the test results are shown in Table 1 and Table 2.

(2) Physical properties of unclassified tailings

The physical properties of unclassified tailings, especially particle size, composition, and density, had a strong influence on the performance and mechanical properties of the CPB. A water sieve method was used to determine the composition of unclassified tailings, and the results are shown in Table 3. Particles less than 74 μm (−200 mesh) accounted for 77.21% of the unclassified tailings, and the content of fine tailings that were less than 37 μm (−400 mesh) reached 50.96%, which are considered fine tailings.

A pycnometer method (YB-373-75) was used to test the true density of the unclassified tailings. The true density of unclassified tailings was 2.658 g/cm^3^.

#### 2.3.2. Slope Stability Research Program

**Figure 4:**
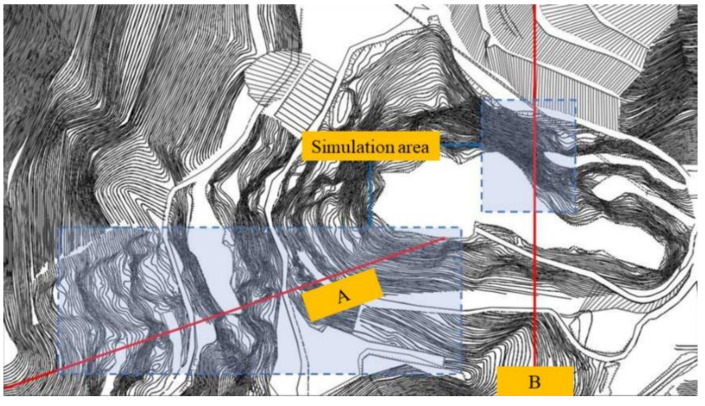
Two slope profiles of the open pit: A, A side slope profile line; B, B side slope profile line.

Two slope profiles were used to investigate slope safety under the influence of the CPB. The locations of the two slope profiles are shown in Figure 4. In this section, the Slide software was used to investigate the safety of open-pit slopes under the influence of the unclassified CPB. The JanBu method based on the optimization non-arc method was used in the simulation.

According to the requirements of Appendix D.2.1 of Technical Code for Slope Engineering of Non-coal Open-pit Mine (GB 51016-2014), when calculating seismic stability, the coefficient of the seismic inertia force of each strip shall be calculated as follows:

(1) $$K_{c}=\alpha \xi \beta_{i}$$

where α is the designed seismic acceleration according to local horizontal seismic coefficients, taken as 0.05; ξ is the reduction coefficient, taken as 0.25; β_i_ is the dynamic distribution coefficient of the i-th block, taken as 1.5 in this stability analysis.

After the calculation, the horizontal seismic inertia force coefficient of the mine design is determined, K_c_ = 0.01875. The seismic inertia force coefficient in the vertical direction is generally 65% of that in the horizontal direction, so 0.0121875 is taken as the value in this study.

#### 2.3.3. Heavy Metals Leaching Experiment

The test method standard for leaching toxicity of solid waste—roll over leaching procedure (GB 5086.1-1997), and solid waste-extraction procedure for leaching toxicity, acetic acid buffer solution method (HJ/T300-2007), were used as the heavy metal leaching methods for tailings and CPB samples.

In the first method, the moisture of each sample was tested with a moisture content meter (Miaozhun Technology (Shenzhen) Co., Ltd., MAY-DS101, Shenzhen, China), and the samples were broken down to below 5 mm in diameter. We weighed 70.0 g of each sample and put it in a 1 L extraction bottle. According to the water content of the sample and the liquid–solid ratio of 10:1 (L/Kg), we calculated the volume of the required extractant, added the extractant, and covered it tightly. After the bottle cap was fixed on the inversion shaking device, the rotation speed was set to 30 ± 2 r/min, and the inversion shaking was carried out at 23 ± 2 °C for 18 h. After the inversion shaking was completed, it was left to stand for 30 min, and the 0.45 μm glass fiber filter membrane was used with a vacuum filter pump. Under these conditions, the leachate was filtered to obtain the final toxic leachate.

In the second method, two extraction agents were used. Reagent #1 was a diluted glacial acetic acid solution with a pH of 4.93 ± 0.05. Reagent #2 was a diluted glacial acetic acid solution with a pH of 2.64 ± 0.05. First, 5.0 g of each sample was placed into a 500 mL beaker or conical flask, 96.5 mL reagent water was added, stirred violently with a magnetic agitator for 5 min, and then the pH was measured. If pH < 5.0, then we used extract #1, if pH > 5.0, then we added 3.5 mL of 1 mol/L HCl, covered the dish, heated it to 50 °C, and kept the solution at this temperature for 10 min. After cooling the solution to room temperature and determining the pH, if the pH < 5.0, we used extract #1, if pH > 5.0, we used extract #2. For the extraction of volatile substances, only extraction reagent #1 was used. The moisture content of the sample was detected using a moisture content tester, and the sample was crushed to less than 0.95 cm. Each 75 g sample was weighed and placed in a 2 L extraction bottle. According to the moisture content of the sample and the liquid–solid ratio of 20:1 (L/Kg), the rotation speed was set to 30 ± 2 r/min, and the inversion shaking was carried out at 23 ± 2 °C for 18 h.

Subsequently, inductively coupled plasma emission spectroscopy (Optima 5300) and flame atomic absorption (HG-9602) were used to analyze the liquid extract [24].

#### 2.3.4. Groundwater Safety Monitoring

**Figure 5:**
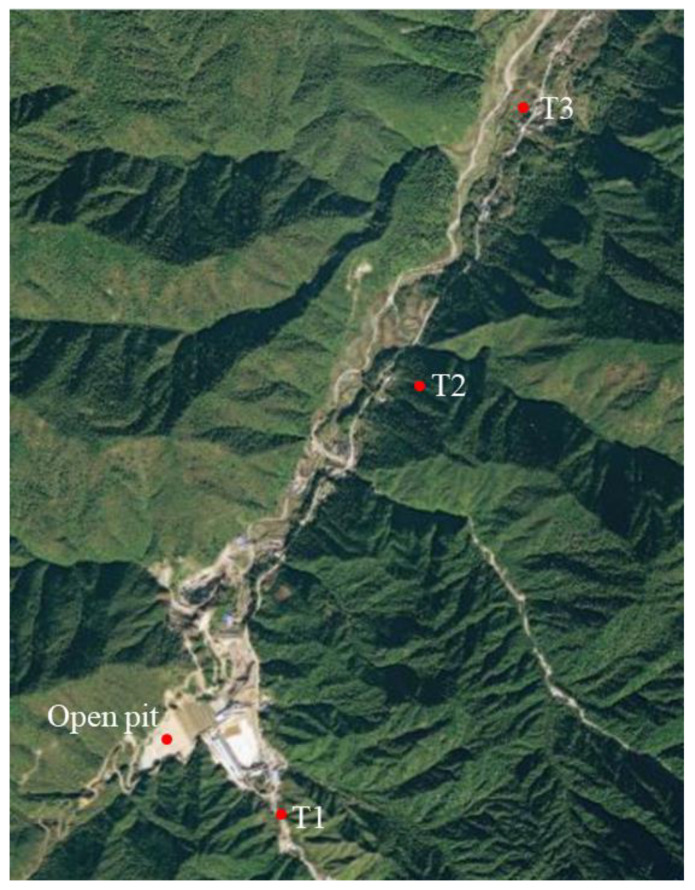
Distribution map of water quality monitoring points.

The mine commissioned an environmental protection and testing company to monitor the groundwater within the affected range of the open pit unclassified CPB project. As shown in Figure 5, the monitoring sites were located in the settlement T1 in the southeast of the mining area, settlement T2 in the northeast of the mining area, and settlement T3 in the northeast of the mining area.

#### 2.3.5. Prediction Method

For the groundwater pollution prediction, the hydrogeological prediction model of pollutant transport in the downstream groundwater is generalized as a one-dimensional stable flow one-dimensional hydrodynamic dispersion problem, and sewage leakage is generalized as a one-dimensional semi-infinite porous medium cylinder of uniform infiltration, with a fixed concentration boundary model at one end:

(2) $$\frac{C}{C_{0}}=\frac{1}{2}\mathrm{erfc}(\frac{x-ut}{2\sqrt{D_{L}t}})+\frac{1}{2}e^{\frac{ux}{D_{L}}}\mathrm{erfc}(\frac{x+ut}{2\sqrt{D_{L}t}})$$

where x is the distance from the injection point, m, from the open pit to the dew point of the downstream spring, predicted every 100 m; t is the time, d, assuming constant contaminant leakage under the most unfavorable conditions; C is the predicted pollutant concentration, g/L; C_0_ is the initial concentration at the pollutant injection point, g/L; u is the flow velocity, m/d; D_L_ is the longitudinal dispersion coefficient, m^2^/d; erfc () is the residual error function.

According to the pumping test results of the zK4/3 hole in the geological report, the permeability coefficient of the karst fissure aquifer can be calculated, K = 0.104 m/d. Then, according to Darcy’s law:

(3) ν=KJ

where *ν* is the seepage velocity of groundwater; J is the hydraulic slope, 10%. The actual flow velocity u of groundwater is calculated as follows:

(4) $$u=\nu /n_{e}$$

where n_e_ is the effective porosity, 0.011.

According to the lithologic characteristics of the prediction area and related stratum research, the dispersion coefficient is calculated using the following empirical formula:

(5) $$D_{L}=0.83\times {\left(\mathrm{lg}L\right)}^{2.414}$$

where L is the approximate maximum inner diameter of the prediction area, approximately 65 m.

## 3. Results and Discussion

### 3.1. The Safety of the Slope

#### 3.1.1. Stability Analysis of Open-Pit Slope without Seepage Field

**Figure 6:**
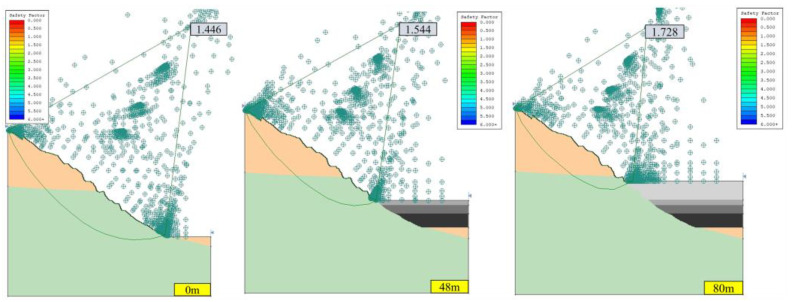
Safety factor of slope A under Ⅰ conditions with different backfilling heights without seepage field (JanBu method).

**Figure 7:**
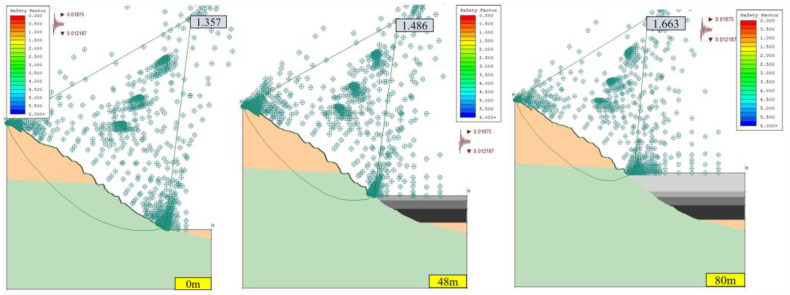
Safety factor of slope A under Ⅱ conditions with different backfilling heights without seepage field (JanBu method).

**Figure 8:**
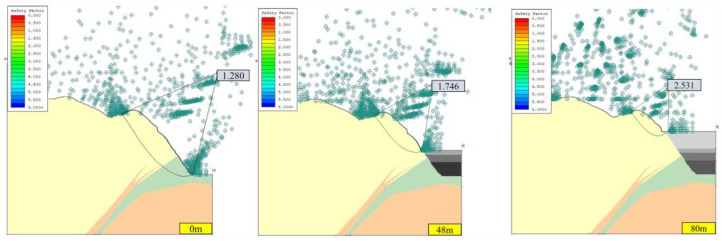
Safety factor of slope B under Ⅰ conditions with different backfilling heights without seepage field (JanBu method).

**Figure 9:**
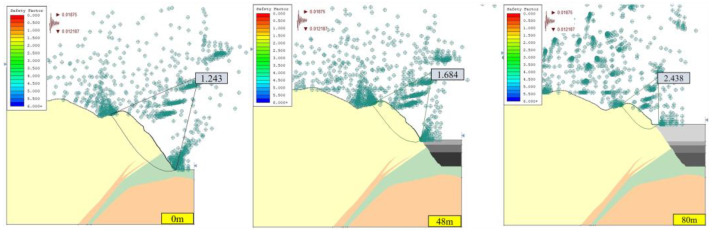
Safety factor of slope B under Ⅱ conditions of different backfilling heights without seepage field (JanBu method).

Under the two working conditions (condition I: dead weight, condition II: dead weight + seismic force) without a seepage field, the calculation results of the safety factor of the open-pit slope in the southwest slope profile (section A) and the north slope profile (section B) are shown in Figure 6, Figure 7, Figure 8 and Figure 9. According to the technical code for no-coal open-pit mine slope engineering (GB 51016-2014), the safety grade of the slope is I. Based on the principle of safety first, the limit safety factor of the slope under condition I is 1.25, and the limit safety factor of the slope under condition II is 1.20.

The slope safety factor is proportional to the height of the landfill; that is, with the increase in the height of the landfill, the slope safety factor gradually increases. When the optimized non-circular JanBu method is adopted, the corresponding safety factors of section B slope are 1.243, 1.684, and 2.438 under working condition II, when the backfilling heights were 0, 48, and 80 m. The corresponding safety factors of section A slope are 1.357, 1.486, and 1.663. This indicates that the consolidation of open pit filling is beneficial to the stability of the slope and can reduce the geological hazards of a large open pit slope to a certain extent. The main reason is that cemented backfill of an open pit and consolidation of the filling body act as a retaining wall, hindering further deformation of the slope [8].

As shown in Figure 6, Figure 7, Figure 8 and Figure 9, the safety factor of slope B is lower than that of slope A, mainly because slope A has a lower angle, while slope B has a higher and steeper angle. Under condition I, the safety factors of slopes A and B meet the requirements of the standard slope safety system (GB-51016-2014 Technical Specifications for Slope Engineering of Non-coal Open-pit Mine). Under condition II, the safety factors of slopes A and B decrease due to earthquakes, but the safety factors of the slopes are all higher than the minimum safety factors.

#### 3.1.2. Stability Analysis of Open-Pit Slope under Seepage Field

**Figure 10:**
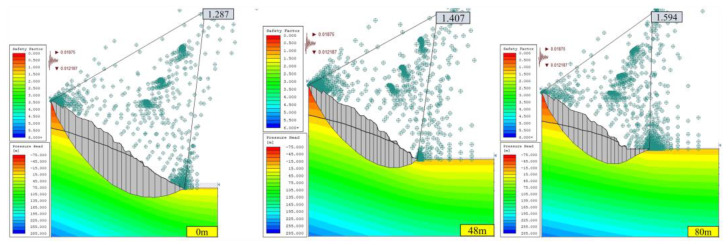
Safety factor of slope in section A under special conditions of different backfilling heights with seepage field (JanBu method).

**Figure 11:**
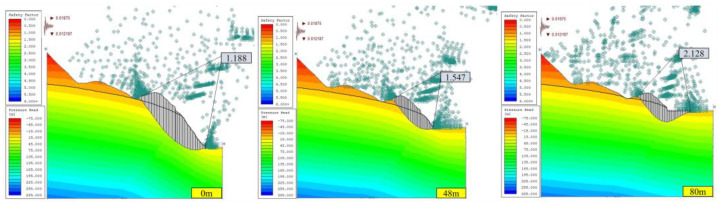
Safety factor of slope in section B under special conditions of different backfilling heights with seepage field (JanBu method).

Considering the effect of the seepage field, the calculation results of the safety factor of the open-pit slope corresponding to different consolidated landfill heights (0, 48, and 80 m) under two working conditions (condition I: dead weight, condition II: dead weight + seismic force) are shown in Figure 10 and Figure 11.

Under condition I with a seepage field, rainwater and groundwater will reduce the safety factor of the slope, mainly because the rainwater and groundwater invading the rock mass can lead to saturation and reduce the cohesive force of the rock mass. Increased fissure water pressure in rock and soil mass, increased density of rock and soil mass, and reduced cohesive forces of the rock mass can occur, which changes the slope’s limit equilibrium state [25].

Under condition II with a seepage field, the safety factor of slope B without backfilling is 1.188, which is lower than the standard safety factor of 1.20 and easily allows natural hazards to occur, such as landslides. Because the seismic loading can reduce the strength of rock and soil [25]. However, with the increase in backfilling height of the open pit, the safety factor of slope B increases gradually, which exceeds the standard safety factor, improves stability, and reduces the probability of natural disasters. The main reason is that the backfilling body has a certain strength and acts as the slope retaining wall, providing the slope with an anti-sliding force, thus improving the safety factor of the slope.

Therefore, under the consideration of seepage fields, such as rainwater and groundwater, slope A can be categorized as a safe slope before the cementation backfill operation (backfilling height of 0 m), while slope B has certain risks of landslide and collapse, but its stability improves with the increase of the backfill height. When the open pit was backfilled, the safety factor can meet the code requirements.

### 3.2. Leaching Properties

**Figure 12:**
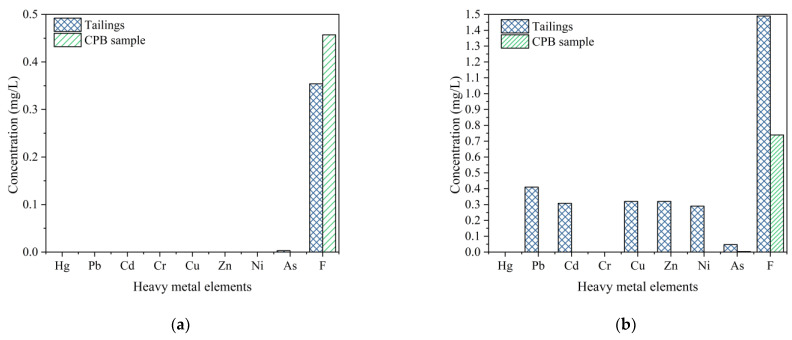
The heavy leaching results of tailings and CPB samples: (**a**) GB5086.1-1997 method, (**b**) HJ/T300-2007 method.

According to the mineral composition and chemical composition of tailings and cement, the pH and amounts of Hg, Pb, Cd, Cr, Cu, Zn, Ni, As, and F were tested using the heavy metal leaching methods. The test results are shown in Figure 12.

As shown in Figure 12, under the condition of GB5086.1-1997, the pH value of unclassified tailings leaching solution (6.4) was slightly lower than the standard limit of class III groundwater (6.5 ≤ pH ≤ 8.5), but the pH value of CPB samples reached 8.37, which met the limit requirements. Under the condition of HJ/T300-2007, the leaching concentration of Pb, Cd, Cu, Ni, As, and F were 0.41, 0.308, 0.32, 0.29, 0.0471, and 1.49 mg/L, respectively. According to the standard of groundwater quality (GB/T14848-2017), the concentration of Pb, Cd, Cu, Ni, As, and F all exceed the groundwater class III limit requirements.

Compared with the unclassified tailings leaching results, all the heavy metal leaching concentrations of the CPB sample were decreased, which indicated that the unclassified tailings CPB played a direct role in the capture of toxic components in tailings under the action of cementing materials [24]. This result is mainly due to the hydration of cement, such that a large number of hydration products were generated, which improves the strength of the CPB body and reduced the leaching concentration of heavy metals [26].

The quality indexes of the leachate of cemented tailings backfill under acid leaching and water leaching conditions all meet the requirements of the class III limit of groundwater. Due to the use of reliable anti-seepage measures, the water in the open pit will be limited to the pit and will not pollute the groundwater environment. The water discharged from the open pit will enter the mine sewage treatment system and be used for operation or discharged after treatment, without causing pollution to the surface water system.

### 3.3. Groundwater Safety Monitoring

To study the influence of cemented backfilling more comprehensively, particularly the effects on groundwater quality, three points were monitored around the open pits, and the groundwater monitoring results are shown in Table 4.

According to the standard of groundwater quality (GB/T14848-2017), all monitoring indexes met the requirements of class III water quality standard, indicating that under the condition of seepage prevention and drainage measures and normal operation of the sewage treatment system, the groundwater environmental quality in this area was good, and the impact of cemented backfilling on the regional groundwater environment is sufficiently small.

### 3.4. Groundwater Safety Monitoring

The results of water quality monitoring at the initial stage of the CPB showed that OPUCPB will not affect the groundwater environment. However, the uncemented backfill of the unclassified tailings is adopted in the extreme case of sudden failure of the backfilling system. In this limit state, the diffusion degree of heavy metals in tailings in groundwater needs to be studied.

**Figure 13:**
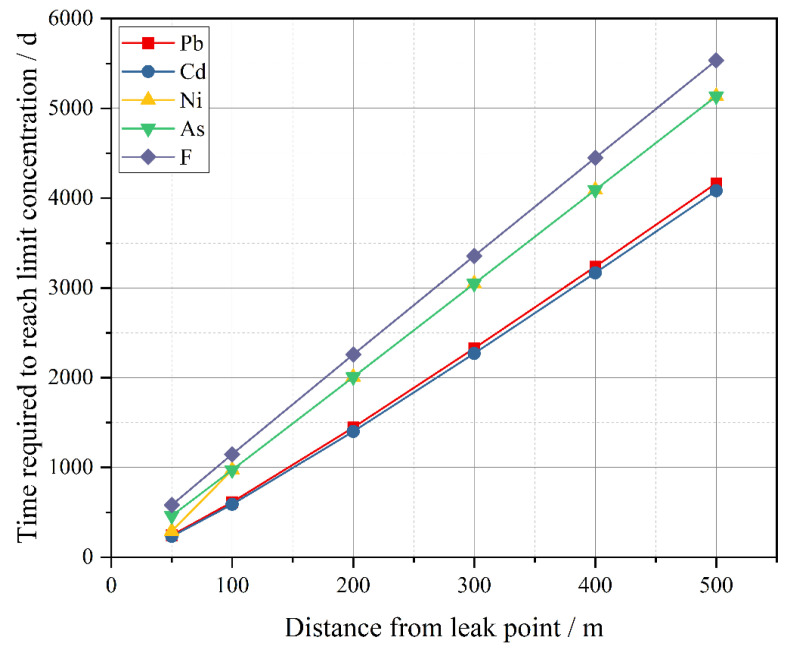
The time curve of heavy metals reaching the limit concentration of class III standard.

According to the heavy metal leaching concentration of tailings under the condition of HJ/T300-2007, the main heavy metals used to predict groundwater pollution are Pb, Cd, Ni, As and F and the initial concentrations used in this report were 0.41, 0.308, 0.29, 0.0471, and 1.49 mg/L, respectively. The prediction points were set at 50, 100, 200, 300, 400, and 500 m from the leakage point, respectively. The results are shown in Figure 13.

Even under extreme conditions, the pollution of groundwater by pollutants through infiltration and underground runoff is a long and slow process. When the predicted point is 50 m away from the leakage point, the minimum time required for the five pollutants to reach the standard limit is also 232 d. When the predicted point is 500 m away from the leakage point, the longest time required for pollutants to reach the standard limit amounted to 5534 d.

With the increase in the distance between the predicted point and the leak point, the time for the pollutant to reach the standard limit concentration shows a linear increasing trend. Under the same distance of the five predicted pollutants, the time required for cadmium, lead, nickel, arsenic, and fluoride to reach the standard limit concentration increases in turn, indicating that the pollution risk also increases correspondingly.

## 4. Conclusions

OPUCPB was initially developed as a response to tougher examination and approval of the tailings and the motivation for the application of new technology, essentially, to ensure the safety of open-pit slopes and environmental protection. The characteristics of OPUCPB were analyzed in this report using slope safety simulations, heavy metal leaching analysis, groundwater monitoring, and pollutant diffusion predictions. The main research results and conclusions of the project can be expressed as follows:

(1) The safety factor of the open pit slope was proportional to the height of OPUCPB. Under the conditions of seismic forces and seepage fields, the safety factor of slope B was increased from 1.188 to 1.574 using OPUCPB.
(2) According to the results of acid and water leaching of tailings and backfilling cement bodies, CPB can effectively capture and fix the heavy metals so that the leaching concentration in tailings changes from exceeding the groundwater class III standard to meeting the standard.
(3) The groundwater monitoring results showed that the groundwater around the open pit conformed to the national groundwater class III limit requirements and OPUCPB had no significant impact on the groundwater.
(4) Considering extreme conditions, a failure of the cementitious material conveying system occurs, and a small number of tailings enter the open pit without consolidation in a short time, thus polluting the groundwater. It was found that the pollution process was very slow, even just 50 m away from the leak point, and the minimum time required for pollutants to reach the standard limit was 232 d. Therefore, the long-term influence of tailings solidification by backfilling in an open pit on the groundwater environment can be controlled. In addition, the heavy metal diffusion in the groundwater should be investigate in the future.

## Figures and Tables

**Table 1:** Physical and mechanical properties of CPB.

Cement-Tailings Ratio	γ (t/m^3^)	Rc (MPa)	Rt (MPa)	C (MPa)	φ (º)	K (m/s)
1:10	1.67	2.38	0.056	0.37	72.56	0.8 × 10^−4^
1:14	1.64	1.67	0.048	0.28	70.76	1.0 × 10^−4^
1:16	1.66	1.52	0.041	0.25	70.69	1.1 × 10^−4^
1:20	1.70	0.88	0.036	0.18	67.13	1.3 × 10^−4^

**Table 2:** Physical and mechanical properties of the rock mass.

Material	γ (t/m^3^)	Rc (MPa)	C/MPa	φ (º)	K (m/s)
Metamorphic Quartz Sandstone	26.46	145.64	44.00	27.84	1.86 × 10^−4^
Fine-grained Granite	25.48	99.68	27.46	32.24	6.59 × 10^−6^
Marble	28.59	88.65	22.79	35.66	3.47 × 10^−6^

**Table 3:** The particle size distribution result from unclassified tailings in CTM.

Particle Size (μm)	+150	−150~+74	−74~+45	−45~+37	−37	Recovery Percent
Content (%)	10.82	11.97	19.37	6.88	50.96	100%

**Table 4:** Summary table of groundwater quality testing results.

Test Items	T1/mg·L^−1^			T2/mg·L^−1^			T3/mg·L^−1^		
	D1	D2	D3	D1	D2	D3	D1	D2	D3
PH	6.21	6.22	6.24	6.31	6.35	6.33	7.36	7.36	7.38
Cu	<0.001	<0.001	<0.001	<0.001	<0.001	<0.001	<0.001	<0.001	<0.001
Pb	<0.001	<0.001	<0.001	<0.001	<0.001	<0.001	<0.001	<0.001	<0.001
Zn	0.09	0.12	0.11	0.11	0.11	0.1	0.1	0.1	0.1
Cd	<0.001	<0.001	<0.001	<0.001	<0.001	<0.001	<0.001	<0.001	<0.001
As	<0.001	<0.001	<0.001	<0.001	<0.001	<0.001	<0.001	<0.001	<0.001
Mn	<0.001	<0.001	<0.001	<0.001	<0.001	<0.001	<0.001	<0.001	<0.001
Fe	<0.001	<0.001	<0.001	<0.001	<0.001	<0.001	<0.001	<0.001	<0.001

## Data Availability

Not applicable.
